# Isolated Congenital Anosmia and CNGA2 Mutation

**DOI:** 10.1038/s41598-017-02947-y

**Published:** 2017-06-01

**Authors:** M. Reza Sailani, Inlora Jingga, Seyed Hashem MirMazlomi, Fatemeh Bitarafan, Jonathan A. Bernstein, Michael P. Snyder, Masoud Garshasbi

**Affiliations:** 1 Department of Genetics, Stanford University, Stanford, CA USA; 2 Medical Genetics Department, DeNA laboratory, Tehran, Iran; 3 Department of Pediatrics, Stanford University, Stanford, CA USA; 4 Department of Medical Genetics, Tarbiat Modares University, Teheran, Iran

## Abstract

Isolated congenital anosmia (ICA) is a rare condition that is associated with life-long inability to smell. Here we report a genetic characterization of a large Iranian family segregating ICA. Whole exome sequencing in five affected family members and five healthy members revealed a stop gain mutation in *CNGA2* (OMIM 300338) (chrX:150,911,102; CNGA2. c.577C > T; p.Arg193*). The mutation segregates in an X-linked pattern, as all the affected family members are hemizygotes, whereas healthy family members are either heterozygote or homozygote for the reference allele. *cnga2* knockout mice are congenitally anosmic and have abnormal olfactory system physiology, additionally Karstensen *et al*. recently reported two anosmic brothers sharing a *CNGA2* truncating variant. Our study in concert with these findings provides strong support for role of *CNGA2* gene with pathogenicity of ICA in humans. Together, these results indicate that mutations in key olfactory signaling pathway genes are responsible for human disease.

## Introduction

Isolated congenital anosmia (OMIM 107200) is a rare condition where patients have no recollection of ever being able to smell^1^. Unlike syndromic forms of anosmia, ICA patients have no additional symptoms, and no other underlying disease-causing condition can be identified^1^. ICA usually occurs sporadically, although some familial cases have previously been reported^1–6^. While several causal genes have been identified for syndromic cases of anosmia (such as Kallmann syndrome and Bardet-Biedl syndrome)^7–9^, the underlying genetic architecture of ICA is barely known. Since Glaser *et al*. first reported ICA as a potentially hereditary trait nearly 100 years ago^10^, there have been few candidate genes identified as the potential genetic cause for ICA^3, 6, 11–13^. Moreover, most reported anosmia cases are inherited through autosomal dominant patterns^2, 14^, however, in some instances, it is an X-linked trait^3, 6, 10^. For instance, In 2015, Karstensen *et al*. for the first time reported a hemizygous nonsense mutation in *CNGA2* in an ICA family (two brothers) with an X-linked pattern of inheritance^3^, but the lack of additional family members precluded definitive assignment of this gene as associated with this disease. *CNGA2* encodes the alpha subunit of a cyclic nucleotide-gated olfactory channel. Interestingly, deletion of any of the three main olfactory transduction components in mice, namely *Gnal* ^15^, *Adcy3* ^16^ and *Cnga2* ^17^, causes significant reduction of physiological responses to odorants consistent with ICA phenotype. Also, Alkelai *et al*.^6^ reported a family with congenital general anosmia segregating an X-linked missense mutation in the *TENM1* gene^6^. Together, these studies indicate that ICA is very heterogeneous and that rare pathogenic variants in multiple genes may contribute to the phenotype.

Here, we report exome-sequencing results of a large consanguineous Iranian family segregating ICA in an X-linked pattern of inheritance. We identified a novel stop-gain mutation in *CNGA2* gene that in concert with Cnga2 knockout mice and results of the Karstensen *et al*. study^3^ further supports the role of *CNGA2* in the pathogenesis of ICA in humans.

## Results

### Exome sequencing results

**Figure 1:**
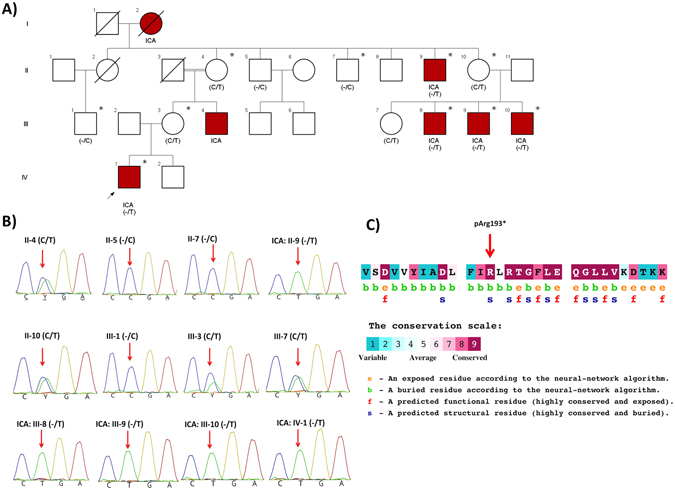
(**A**) Pedigree structure of ICA family. The proband is noted by an arrow. Family members for which whole exome sequencing has been performed are noted by a star above the individual’s symbol. The genotype of identified variant for family members for which DNA sample was available is mentioned below the individual’s symbol. (**B**) Sanger sequencing traces (C**C/T**GA) showing the c.950 C > T (p.Arg193*) mutation in exon 6 of the *CNGA2* gene. The segregation of this mutation has been confirmed in 12 available DNA samples (Five affected and seven unaffected individuals) from this family. (**C**) The amino-acid sequence CNGA2 (pArg193*) colored according to the conservation scores. Conservation scores were calculated by ConSurf tool^18^. ConSurf estimates the evolutionary conservation of amino acid residues in a peptide based on the phylogenetic relations between homologous sequences as well as amino acid’s structural and functional importance.

Whole-exome sequencing to a mean coverage of above 70X was performed (Supplemental Fig. 1). Using Varseq software, we identified 88,042 variants shared in the all sequenced family members (10 individuals; Table 1) that have a genotype quality score above 20. Since the disease model of ICA based on the pedigree was consistent with autosomal recessive or X-linked, we filtered for homozygous or hemizygous variants that are found in all the five affected individuals, but in none of the healthy controls. 16 variants passed this filtering step of which only CNGA2.577C > T has minor allele frequency (MAF) less than 0.01 or absent in the public database (dbSNP Common 144 (Database of Single Nucleotide Polymorphism, NCBI), 1000 Genome project phase 3 (www.1000genomes.org), Exome Aggregation Consortium version 0.3 (EXaC), Exome Variant Server; NHLBI GO Exome Sequencing Project (ESP), Seattle, WA (http://evs.gs.washington.edu/EVS/), and the Iranian genome project (http://irangenes.com/data-2/). This loss of function mutation occurs in exon 6 of *CNGA2* gene, is rare (unreported in public databases) and highly conserved (Fig. 1C) by *ConSurf* ^18^.

**Table 1:** ICA Family members ID and variants filtering steps.

Family ID	II-4	II-5	II-7	II-9	II-10	III-1	III-3	III-7	III-8	III-9	III-10	IV-1
**Age**	53Y	60Y	63Y	51Y	52Y	35Y	41Y	34Y	32Y	28Y	22Y	16Y
**Sex**	female	male	male	male	female	male	female	female	male	male	male	male
**Disease status**	Healthy	Healthy	Healthy	ICA	Healthy	Healthy	Healthy	Healthy	ICA	ICA	ICA	ICA
Smell test results*	NA	NA	NA	0/24	19/24	NA	20/24	20/24	0/24	7/24	0/24	0/24
**Total Variants**	114760	108192	NA	113826	112106	108115	111921	NA	112019	113508	111772	112236
**Shared variants**	88,042											
**Homozygous variants**	16 (Homozygous in affected members, but either heterozygous or homozygous for reference allele in controls)											
**1KG MAF** < **0.01**	1											
**EXaC MAF** < **0.01**	1											
**dbSNP 144 MAF** < **0.01**	1											
**NHLBI MAF** < **0.01**	1											
**Exonic Variants**	1											
**Candidate**	chrX:150,911,102; CNGA2.aAug10:c.577 C > T; p.Arg193*											

Y, year. NA, Not Available. ICA, Isolated Congenital Anosmia. *Based on Iran Smell Test. MAF, minor allele frequency. 1 KG, 1000 Genome project phase 3,. EXaC, Exome Aggregation Consortium version 0.3. dbSNP 144, Database of Single Nucleotide Polymorphism, NCBI. NHLBI, Exome Variant Server; NHLBI GO Exome Sequencing Project (ESP).

### Segregation analyses

We confirmed this variant by Sanger sequencing (Fig. 1B) and showed that the variant co-segregates with the disease as all the five affected individuals have a hemizygous mutation (one mutation on the X chromosome in males), while unaffected family members are heterozygous or homozygous for the reference allele.

## Discussion

Anosmia occurs mostly in the middle aged and elderly; affecting up to 5% of individuals over the age of 45 years old^19, 20^. Rarely, however, it can occur as a congenital condition (prevalence 1 in 10,000)^21^ (Isolated Congenital Anosmia) (Online Mendelian Inheritance in Man [OMIM] % 107200). Few candidate genes have been identified as potential causal genes for ICA in humans^3, 6, 11–13, 22^. For instance, Karstensen *et al*.^3^ reported a mutation in *CNGA2* in two brothers with an X-linked pattern of ICA^3^. However, the lack of additional family members precluded definitive assignment of this gene as associated with this disease. Also, Alkelai *et al*.^6^ reported a family with congenital general anosmia segregating an X-linked missense mutation in the *TENM1* gene^6^. In the present study, we employed whole-exome sequencing to identify the underlying genetic variants associated with ICA in a large Iranian family (five affected and five un-affected family members). The identified loss of function variant has not been reported in the public databases and falls within exon 6 of *CNGA2* gene. We confirmed the segregation of the variant with Sanger sequencing and showed all five affected individuals are hemizygous for this variant, whereas all healthy individuals are either heterozygous or homozygous for the reference allele. Furthermore, one may speculate that the healthy female individuals, who carry this mutation, show to some extent hyposmia. However, the results of smell test on the three apparently healthy females, who carry CNGA2 mutation as well, show that they fall within normosmia category (score of 19 to 24 of smell test) (Table 1). Moreover, due to the lack of brain MRI data, we cannot verify whether there is a defect in olfactory structures (i.e. olfactory bulb and olfactory sulci) in the affected individuals. There have been also contradicting reports on the ICA characterization in humans. Generally ICA is characterized by the absence or severe hypoplasia of olfactory bulbs^23–26^, however, brain MRI studies of ICA patients by Karstensen *et al*.^3^ and Ghadami *et al*. 2004 show that there is an apparently normal or slightly reduced olfactory structure in ICA. These studies suggest the existence of an ICA sub-phenotype characterized by an apparently normal olfactory structure^3, 5^.

Olfactory receptors are the largest group of orphan G protein-coupled receptors^27^. The CNG olfactory channels are made of alpha subunit and two modulatory subunits, encoded by CNGA2, CNGB1b and CNGA4, respectively^28, 29^. The alpha subunit of CNG channel is critical for olfactory sensory neurons to generate an odor-induced action potential. Knockout mice models lacking these essential CNG channel subunits strongly support the critical role of this canonical pathway in olfactory signaling^15–17^. Cnga2 knockout mice are congenitally anosmic and have severely impaired olfactory function^17^. Moreover, CNGA2 is highly expressed in the olfactory sensory neurons of zebrafish, mouse and humans^30–32^. Our study together with Karstensen *et al*.^3^ and Cnga2 knock-out mice model strongly support the role of CNGA2 gene in pathogenicity of familial cases of ICA. Together, these results indicate that mutations in key olfactory signaling pathway genes are responsible for human disease.

## Material and Methods

### Family description

All participants, or their legal guardian, provided written and informed consent. The institutional review boards of Department of Medical Genetics, Tarbiat Modares University, and Stanford University reviewed and approved the project. All methods in this study were performed in accordance with the approved guidelines and regulations. The institutional review board of Stanford University approved the experimental protocols. The family pedigree is shown in Fig. 1A. All the affected individuals underwent clinical and physical examination at the Department of Medical Genetics, DeNA Laboratory, Tehran, Iran.

For assessment of olfactory function in affected individuals, we used Iran Smell Identification Test (Iran-SIT)^33^ which is a commercially Iranian adopted version of the University of Pennsylvania Smell Identification Test (UPSIT)^34^. In this test, the familiar smells in Iranian environment and culture is used to test the function of an individual’s olfactory function in a scale of 1 to 24. A score of 1–9 indicates anosmic; 9–13 severe microsmia; 13–17 mild microsmia, and 19–24 normosmia state (Table 1). The smell test has been performed on all the available affected individuals. For the apparently healthy individuals, smell test has been done only on the three available individuals (II-10, III-3 and III-7 of Table 1 and Fig. 1A).

### Exome sequencing and variants calling

Library preparation was performed using KAPA HyperPlus kit followed by exome capture using the IDT xGen® Exome Research Panel v1.0. Briefly, 0.4 μg of gDNA was fragmented to a peak size of 150–200 bp using the KAPA Frag enzyme. The fragmented genomic DNA were end-repaired, A-tailed, ligated to indexing-specific paired-end adaptors and PCR amplified according to manufacturer’s instructions. The PCR reactions were cleaned using the Agencourt AMPure XP beads. To capture exonic regions, 500 ng of pooled libraries (8 libraries per pool) was hybridized to biotinylated oligonucleotides for 4 hours at 65 °C. The captured libraries were pulled down using Dynabeads MyOne Streptavidin M-270 (Invitrogen). A post-capture PCR was then performed to amplify the captured libraries and to add the barcode sequences for multiplex sequencing for 12 cycles. Afterwards, amplified libraries were purified with AmpPure XP Beads. Qubit fluorometer and Bioanalyzer high sensitivity chips were used to determine the final concentration of each captured library. The pooled libraries were paired-end sequenced on two lane of Illumina HiSeq4000 at the Stanford Center for Genomics and Personalized Medicine according to standard protocols.

### Bioinformatics analyses

DNA libraries were processed and analyzed using the Roche Sequencing Solutions (Bina Technologies version 2.7.9) whole-exome analysis workflow with default settings. Briefly, libraries were mapped with BWA mem 0.7.5 software to human genome (hg19 version), and then realigned around indels with GATK IndelRealigner. Next, base recalibration was performed with GATK BaseRecalibrator taking into account the read group, quality scores, and cycle and context covariates. Variants were called with GATK HaplotypeCaller with the parameters–variant_index_ type LINEAR–variant_index_parameter 128000. VQSR was used to recalibrate the variants, first with GATK VariantRecalibrator and then ApplyRecalibration. Variant filtering and annotation was done using Golden Helix VarSeq Version 1.1 software (http://goldenhelix.com/products/VarSeq/). After importing the variant call files (gVCF files) of each member of the family, the variants were organized by pedigree. Using the 1000 genomes variant frequencies (phase 1), the Exome Aggregation Consortium (ExAC) variant frequency database version 0.3 (Cambridge, MA), and the NHLBI Exome Sequencing Project (https://esp.gs.washington.edu/drupal/) V2 Exome Variant Frequencies, rare variants (minor allele frequency <1%) were filtered. Variants were then classified according to whether they were deemed to be coding.

### Sanger sequencing

To validate the variant and to verify its segregation in the family, we used 5′ TCTACATTGCGGACCTCTTC3′ as forward primer and 5′ TCTAAGAGAACACCCCGAGA3′ as reverse primer for PCR amplification of the variant sequence. PCR amplification was performed using following reagents: REDTaq ReadyMix PCR Reaction Mix (Sigma-Aldrich, St. Louis) 25 ul, 1 ul forward primer (10 uM), 1 ul reverse primer (10 uM), 1 ul DNA (50 ng/ul), and 22 ul of water per PCR reaction. An initial denaturation step at 94° for 3 min was followed by 35 cycles of 94° for 30 sec, 57° for 30 sec, 72° for 30 sec, and the process completed by a final extension at 72° for 7 min. The PCR amplification resulted in a single DNA band on a standard 1% Agarose Gel, and was purified by Agencourt AMPure XP beads (Beckman Coulter, Inc) before submitting for Sanger sequencing. The reverse primer 5′ TCTAAGAGAACACCCCGAGA 3′ was used as sequencing primer. Sanger sequencing was carried out by the Stanford PAN facility using ABI 3130xl Genetic Analyzer.

## Electronic supplementary material

Figure S1
