# Supplementary material for: Isolated Congenital Anosmia and CNGA2 Mutation
Source: Sci Rep. 2017 Jun 1;7:2667. doi: 10.1038/s41598-017-02947-y (PMC5454015; doi:10.1038/s41598-017-02947-y)

Isolated Congenital Anosmia and CNGA2 Mutation

M.Reza Sailani[1,&], Inlora Jingga[1,&], Seyed Hashem MirMazlomi[2], Fatemeh Bitarafan[2], Jonathan A. Bernstein[3], Michael P. Snyder[*1], Masoud Garshasbi[*2,4]

1. Department of Genetics, Stanford University, Stanford, CA USA
2. Medical Genetics Department, DeNA laboratory, Tehran, Iran
3. Department of Pediatrics, Stanford University, Stanford, CA USA
4. Department of Medical Genetics, TarbiatModares University, Iran.

[&] Equal Contribution

***Corresponding authors:**

Michael. P. Snyder

Department of Genetics, Stanford University, Stanford, CA, USA.

Tel: +1 650 736-8099, E.mail: mpsnyder@stanford.edu

Masoud Garshasbi

Department of Medical Genetics; Faculty of Medical Sciences; TarbiatModares University; Tehran, Iran,

Tel: +98-21-82884569, E.mail: masoud.garshasbi@modares.ac.ir

Supplemental Figure 1. Exome target region coverage.

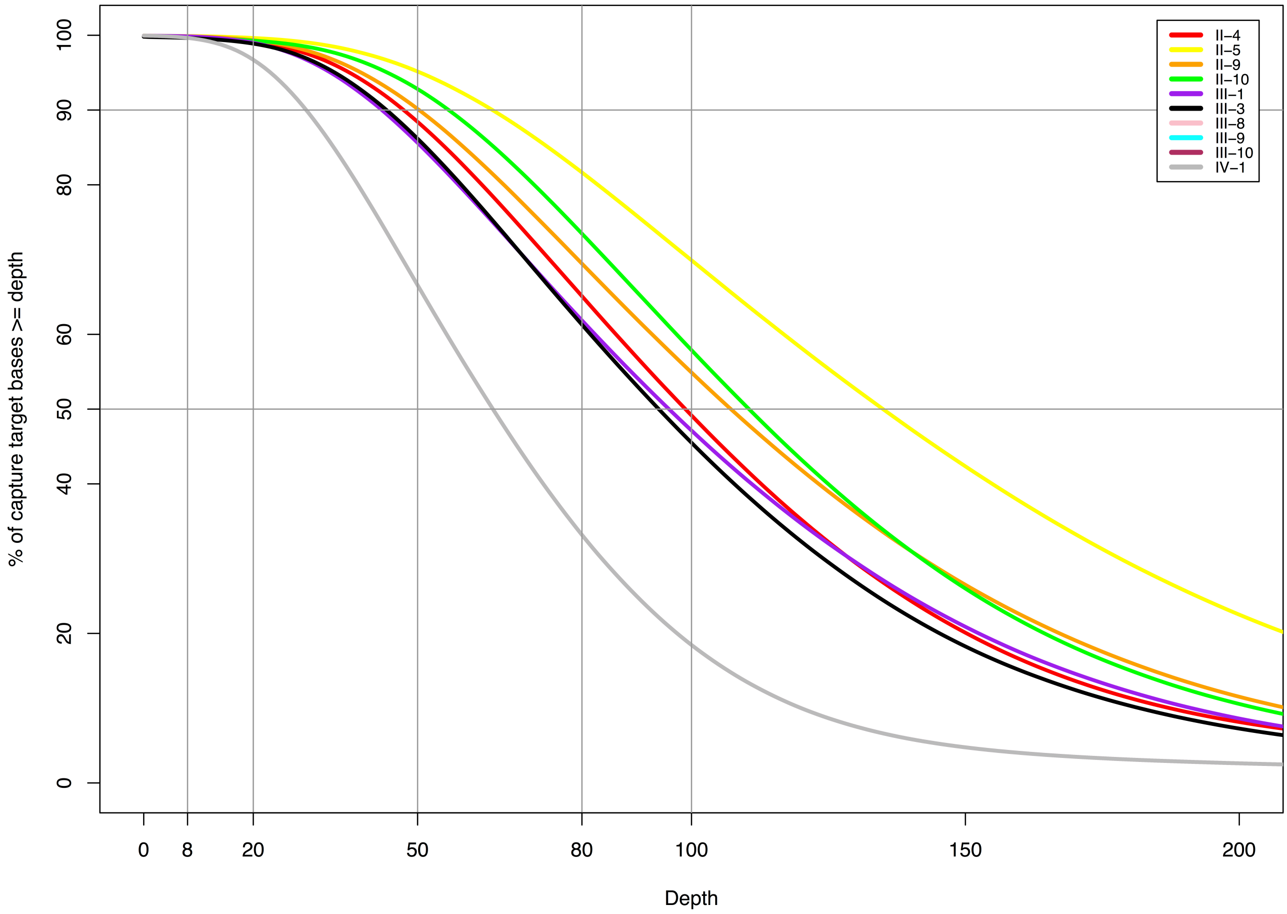

Supplement: Supplementary file 1 — Figure S1 [file 41598_2017_2947_MOESM1_ESM.pdf]
